# Integrated analysis of microRNA and mRNA expression profiles in Preeclampsia

**DOI:** 10.1186/s12920-023-01740-3

**Published:** 2023-12-01

**Authors:** Zepeng Ping, Ying Feng, Ying Lu, Ling Ai, Huling Jiang

**Affiliations:** Department of Obstetrics, Maternity and Child Health Care Affiliated Hospital, Jiaxing University, 2468 Central South Road, Jiaxing, 314000 China

**Keywords:** Preeclampsia, miRNAs, mRNAs, Signaling pathways

## Abstract

**Background:**

Preeclampsia (PE), a pregnancy specific syndrome, is one kind of common gestational hypertension disease, which can cause maternal and perinatal mortality and morbidity. This study was conducted to identify key microRNAs (miRNAs), mRNAs and related signaling pathways in the pathogenesis of PE.

**Methods:**

Whole transcriptome sequencing and small RNA sequencing of the peripheral blood from 3 PE patients and 3 normal pregnant women were performed. Differential expressed (DE) miRNAs were identified using the DEseq2 package. Target genes of the selected upregulated and downregulated DE miRNAs were predicted. Based on the hypergeometric distribution of DE miRNA target genes, we analyzed GO enrichment and KEGG pathway enrichment using R.

**Results:**

Total 1291 and 1281 novel RNAs were obtained from the preeclampsia patients and healthy individuals. 70 miRNAs were screened out with significant levels with 51 significantly upregulated and 19 significantly downregulated. 44,306 genes were predicted as the targets of these miRNAs. Besides, KEGG pathway analysis revealed that the upregulated miRNAs were enriched in Glycosaminoglycan biosynthesis-chondroitin sulfate / dermatan sulfate, Base excision repair and the downregulated miRNAs were enriched in Tuberculosis, Phagosome.

**Conclusion:**

We constructed regulatory networks of miRNAs and target genes, there were 2208 negative miRNA-mRNA interactions in total. The network and pathway information illustrate the potential functions of mRNAs and miRNAs in PE pathogenesis.

## Introduction

Preeclampsia (PE) is a pregnancy associated multisystem disorder after 20 gestational weeks, which characterized by hypertension and proteinuria [3, 12, 33]. It becomes one of the pivotal factors in maternal and neonatal morbidity and arises in 2–8% of pregnancies all over the world [32]. The major pathological mechanism occurs due to inadequate blood perfusion and ischemia, which results from defective placentation resulting to decreased utero-placental perfusion, placental ischemia and dysregulated maternal immune response [7, 21, 29].

MicroRNAs (miRNAs), usually 22–24 nucleotides long, are normally considered as a type of small non-coding RNAs, which endogenous and highly conserved. They combine with specific regions of the 3’-untranslated region (3’‑UTR) of their targets and produce a marked effect to inhibiting the expression of target genes, thus inducing transcript degradation and/or translational suppression [4, 14]. Accumulating evidence have indicated that miRNAs play pivotal regulatory roles in large subsets of biological processes including metabolism, apoptosis, cell proliferation, differentiation and development [13]. Increasing studies have showed large number of miRNAs were associated with the placenta and circulating in preeclampsia [5, 16, 18]. Dong et al. demonstrated that downregulations of circulating miR-31 and miR-21 are associated with preeclampsia [8]. Youssef et al. reported the association of microRNA-210 and microRNA-155 with severity of preeclampsia [37].

In this study, we used transcriptomic to analyze the small RNA expression patterns between individuals with PE and healthy individuals. Meanwhile, potential target genes of DE miRNAs were indicated using TargetScan [10] and miRanda [26] algorithms. Next, Gene Ontology (GO) term and KEGG pathways were conducted to analyze the DE genes to obtain the relation of mRNAs and miRNAs in pathogenesis of PE. Furthermore, this illustrates the the miRNA-mRNA regulatory network in the PE. Overall, these findings provide us novel insights into the regulatory mechanisms related to PE pathogenesis.

## Materials & methods

### Patients with preeclampsia & healthy individuals

All the patients were from inpatients in Maternity and Child Health Care Affiliated Hospital, Jiaxing University. In addition, all participants provided their informed consent. The peripheral blood was excised from preeclampsia patients without major complications such as diabetes, primary hypertension, kidney disease and so on. 40 pregnant individuals (20 PE patients and 20 normal pregnant women) were recruited from the Maternity and Child Health Care Affiliated Hospital, Jiaxing University. According to the American College of Obstetricians and Gynecologists’s diagnostic criteria for preeclampsia: systolic blood pressure ≥ 140mmHg or diastolic blood pressure ≥ 90mmHg after 20 weeks of pregnancy, combined with 24-hour urine protein greater than 300 mg. Severe preeclampsia is diagnosed if symptoms such as thrombocytopenia, renal insufficiency, impaired liver function, pulmonary edema, and new-onset headache are combined. We selected the three most severe preeclampsia patients and three paired healthy pregnant women for whole transcriptome sequencing and small RNA sequencing .

### Blood sample collection & RNA extraction

3.0 ml whole blood samples were collected with EDTA anticoagulant from three paired pregnant women. Leukocytes were isolated from whole blood using an extraction kit (Tiangen, Beijing, China) within 4 h. RNAs was extracted from the leukocytes with TRIzol reagent (Invitrogen Carlsbad, CA, USA). RNA integrity and quantification were assessed using an Agilent 2100 Bioanalyzer (Agilent Technologies, CA, USA) and NanoDrop™ ND-2000 (Thermo Fisher Scientific, CA, USA), respectively.

### Whole transcriptome sequencing for mRNA & bioinformatics analysis

Use the TruSeq Stranded Total RNA with Ribo-Zero Gold kit to establish a cRNA library according to the instructions, and after passing the quality test with the Agilent 2100 Bioanalyzer, use the Illumina sequencing platform(HiSeqTM 4000, Illumina, CA, USA) to generate 150 bp/125 bp double-terminal readings. Raw data have been uploaded to Sequence Read Archieve: PRJNA665923. Using Bowtie2 to map readings from six samples to assembled transcripts, gene expression was estimated as fragments per million readings per KB. The DESeq software (http://bioconductor.org/packages/release/bioc/html/DESeq.html) [3] was used to identify the DE genes. mRNAs showing folds change (FC) > 2 with adjusted *P* < 0.05 were considered DE. Hierarchical clustering and heat maps were created using the R platform (http://www.rproject.org/). GO and KEGG analysis of DE mRNAs were conducted by DAVID bioinformatics resources (https://david.ncifcrf.gov/home.jsp).

### Small RNA sequencing & bioinformatic analysis

Total RNAs was extracted from PE and healthy pregnant women blood samples using a TRIzol reagent kit (AppliedBiosystems, p/n AM1556, CA, USA) according to the manufacturer’s protocol. After being purified by QIAGEN RNeasyR Kits (QIAGEN, Mainz, Germany), RNA quality was assessed using an Agilent 2100 Bioanalyzer. Small RNA sequencing was conducted by ShangHai Oebiotech Co. (Shanghai, China). Small RNA sequencings data has been uploaded to Sequence Read Archieve: PRJNA836097. The sequencing reads were aligned and subjected to BLAST (v2.2.28+) search against Rfam (v.10.1) (http://www.sanger.ac.uk/sof tware/Rf am) and GenBank databases. We identified known miRNAs by aligning with the miRBase (v.21) database (http://www.mirbase.org/) and analyzed the known miRNA expression patterns in different samples. We used MiRDeep2 (v.2.0.0.8) to analyze unannotated small RNAs to predict new miRNAs (Zhu et al., 2009). Based on the pre-miRNA hairpin structure and the miRBase database, the corresponding miRNA star sequences were identified. DE miRNAs with *P* value < 0.05 and FC > 2 were identified. P-values were calculated using the DESeq algorithm in the R package for biological replicate experiments. GO enrichment and KEGG pathway enrichment analysis of DE miRNA target genes based on hypergeometric distribution was performed using R (v3.2.0) [19, 20]

### Integration analysis

Targets of DE miRNAs were predicted using TargetScan [10] and miRanda [26] algorithms with parameters: S ≥ 150 G ≤ -30 kcal/mol and requiring strict 5’-seed pairing. R (http://www.R-project.org) was utilize to calculate the Pearson correlation coefficient to determine the negative correlation between the expression levels of each miRNA and its mRNA targets (25). According to the DE miRNAs and corresponding target genes, each miRNA was screened to be negatively correlated with the expression level of its mRNA targets, and Cytoscape (version 3.0.1; http://www.cytoscape.org/) was used to construct the regulatory networks.

## Results

### Patient information comparison & principal component analysis plot

**Fig. 1:**
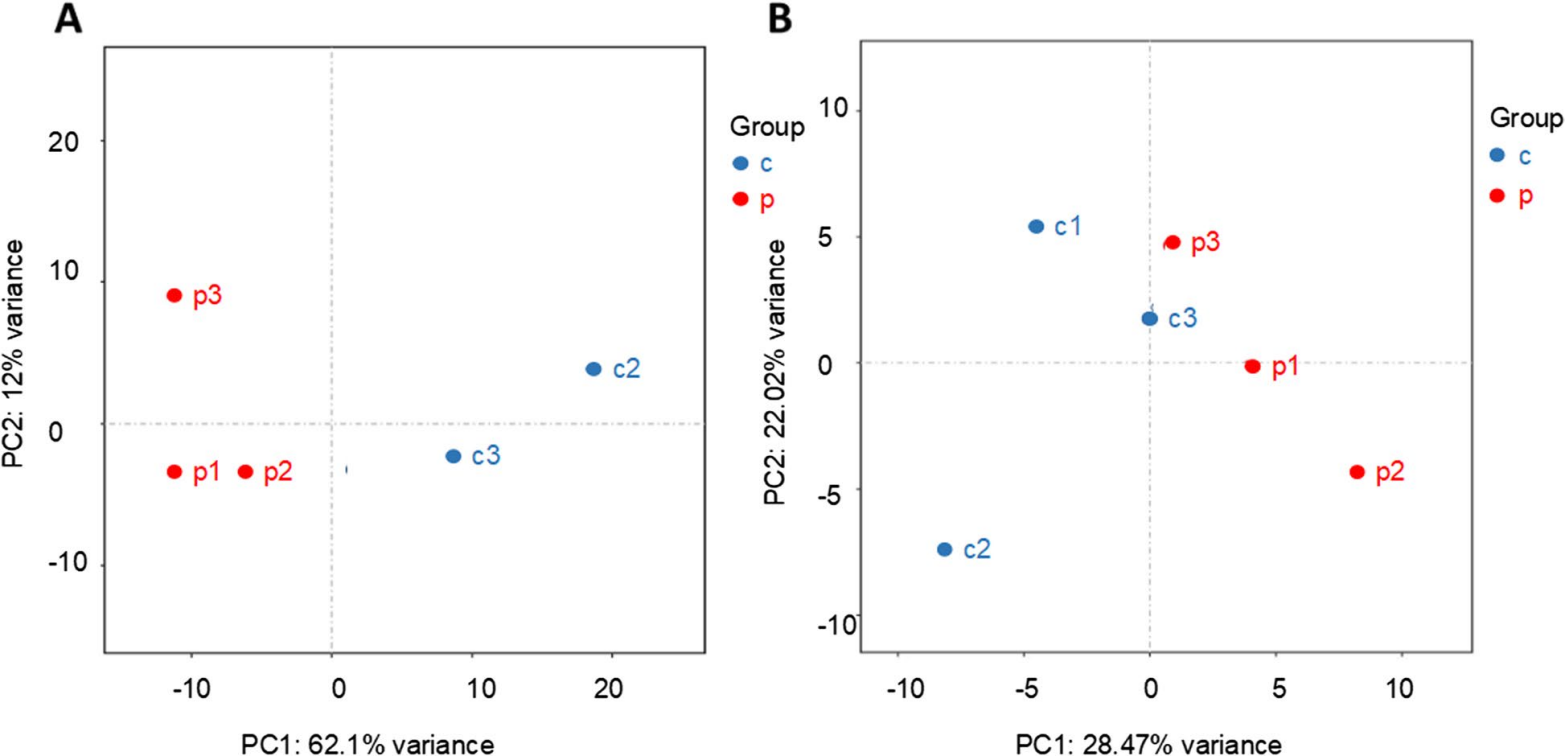
Principal component analysis was performed for mRNA (**A**) and microRNA (**B**)

Basic information was compared for the 20 patients with preeclampsia and 20 healthy control pregnant women. We compared age, gestational age, body mass index(BMI), systolic and diastolic blood pressure, and urine protein quantification in two groups. There were no significant differences in age, gestational age and BMI between the two groups, and there were significant differences in blood pressure and urine protein quantification (Table 1). Prior to the whole transcriptome sequencing and small RNA sequencing, we performed principal component analysis for microRNA and mRNA (Fig. 1).

**Table 1:** The information of patients with PE and normal control groups

Parameter	PE (*n* = 20)	Healthy controls (*n* = 20)	*P* value
Age (years)	27.59 ± 4.20	27.65 ± 2.45	*P*>0.05
Gestational weeks	36.23 ± 2.81	36.88 ± 2.71	*P*>0.05
Body mass index(BMI)	23.15 ± 1.41	22.34 ± 2.05	*P*>0.05
Systolic blood pressure (mmHg)	168.91 ± 13.25	116.55 ± 13.18	*P*<0.01
Diastolic blood pressure (mmHg)	109.91 ± 10.94	69.8 ± 9.89	*P*<0.01
Urine protein (g/24 h)	2.73 ± 1.69	0.09 ± 0.06	*P*<0.01

### mRNA sequencing & analysis

To better understand the pathological mechanism of PE, we performed comparative transcriptomic analysis in 3 PE patients and 3 healthy pregnant women. A total of six cDNA libraries were constructed and sequenced, namedC1, C2, C3, P1, P2 and P3. From libraries; 100,968,860; 84,124,574; 93,671,984; 112,327,098; 116,416,902;107,328,648 clean readings were obtained and 97.47%; 96.00%; 96.81%; 98.27%; 97.65%; 98.28% of the readings were mapped to the reference genome (Table 2).

**Table 2:** Statistical results of reads versus reference genome alignment.(C1, C2, C3,P1, P2 and P3)

Sample	C1	C2	C3	P1	P2	P3
Total reads	100,968,860	84,124,574	93,671,984	112,327,098	116,416,902	107,328,648
Total mapped reads	98,410,469(97.47%)	80,761,974(96.00%)	90,679,812(96.81%)	110,381,982(98.27%)	113,681,744(97.65%)	105,478,039(98.28%)
Multiple mapped	5,517,703(5.46%)	11,864,341(14.10%)	10,680,668(11.40%)	5,618,803(5.00%)	5,839,591(5.02%)	4,908,608(4.57%)
Uniquely mapped	92,892,766(92.00%)	68,897,633(81.90%)	79,999,144(85.40%)	104,763,179(93.27%)	107,842,153(92.63%)	100,569,431(93.70%)
Read-1	46,467,946(46.02%)	34,482,900(40.99%)	40,013,656(42.72%)	52,372,350(46.62%)	53,907,736(46.31%)	50,312,496(46.88%)
Read-2	46,424,820(45.98%)	34,414,733(40.91%)	39,985,488(42.69%)	52,390,829(46.64%)	53,934,417(46.33%)	50,256,935(46.83%)
Reads map to ‘+’	46,449,192(46.00%)	34,436,432(40.94%)	40,026,663(42.73%)	52,388,785(46.64%)	53,946,432(46.34%)	50,299,909(46.87%)
Reads map to ‘-‘	46,443,574(46.00%)	34,461,201(40.96%)	39,972,481(42.67%)	52,374,394(46.63%)	53,895,721(46.30%)	50,269,522(46.84%)
Non-splice reads	73,546,464(72.84%)	55,384,769(65.84%)	63,325,155(67.60%)	85,484,277(76.10%)	89,219,702(76.64%)	79,552,021(74.12%)
Splice reads	19,346,302(19.16%)	13,512,864(16.06%)	16,673,989(17.80%)	19,278,902(17.16%)	18,622,451(16.00%)	21,017,410(19.58%)
Reads mapped in proper pairs	90,007,170(89.14%)	66,460,176(79.00%)	77,202,880(82.42%)	101,626,024(90.47%)	104,133,722(89.45%)	98,012,698(91.32%)

### Identification of DE gene

**Fig. 2:**
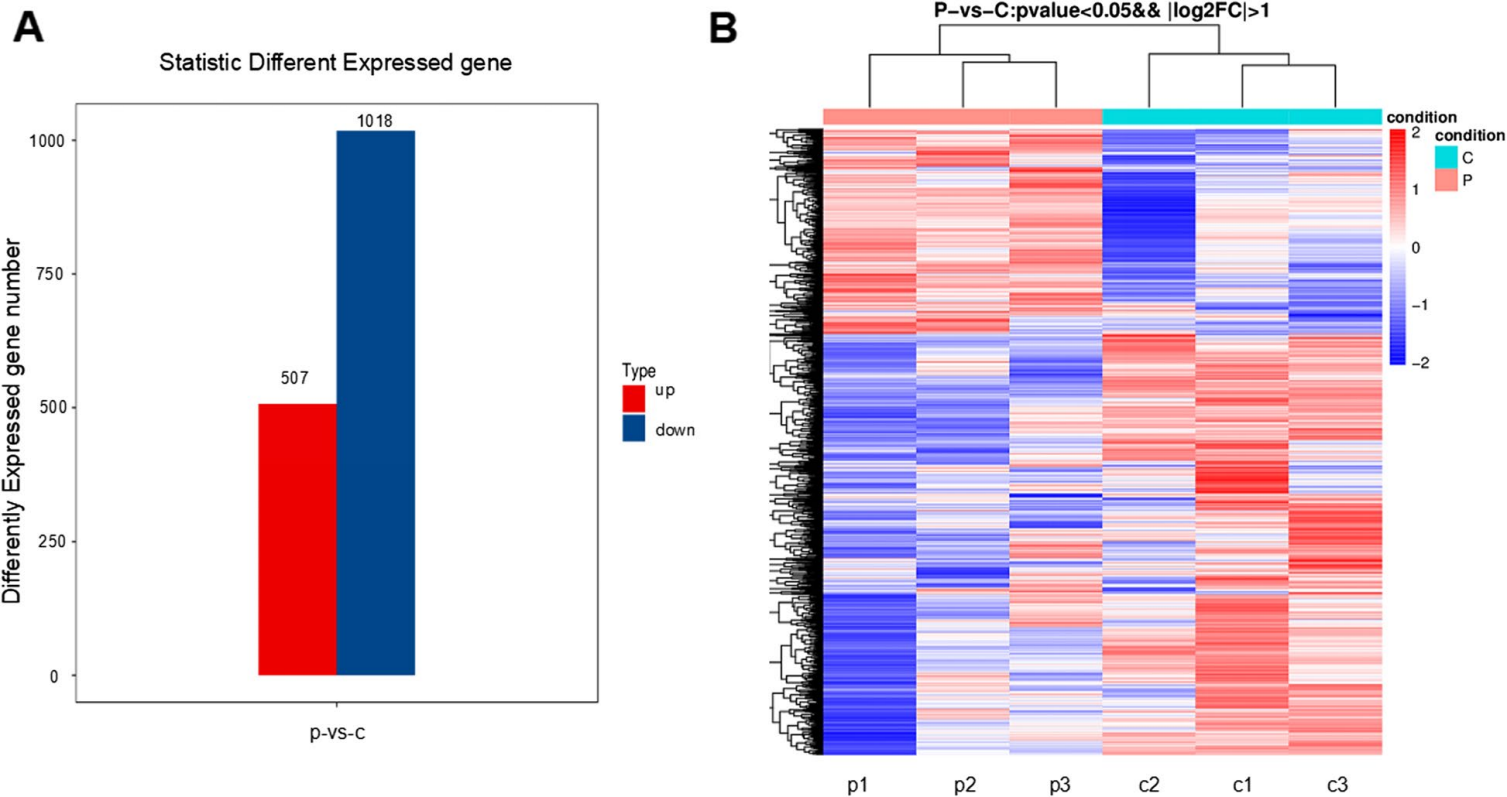
**A** 507 significantly upregulated mRNAs and 1018 significantly downregulated mRNAs between patients with preeclampsia and healthy pregnant women. **B** heat map of differentially expressed mRNAs

Significant differences in mRNA between patients with preeclampsia and healthy pregnant women were screened for fold changes (greater than or equal to twofold changes) and adjusted *p*-values (*p* < 0.05) determined by DESeq analysis. A total of 1525 mRNAs were screened for significantly different expression levels, of which 507 were significantly upregulated and 1018 were significantly downregulated (Fig. 2A). Unsupervised hierarchical clustering of differentially expressed genes generated a heat map of differentially expressed mRNA (Fig. 2B).

### GO term & KEGG pathway enrichment analysis of DE mRNA

**Fig. 3:**
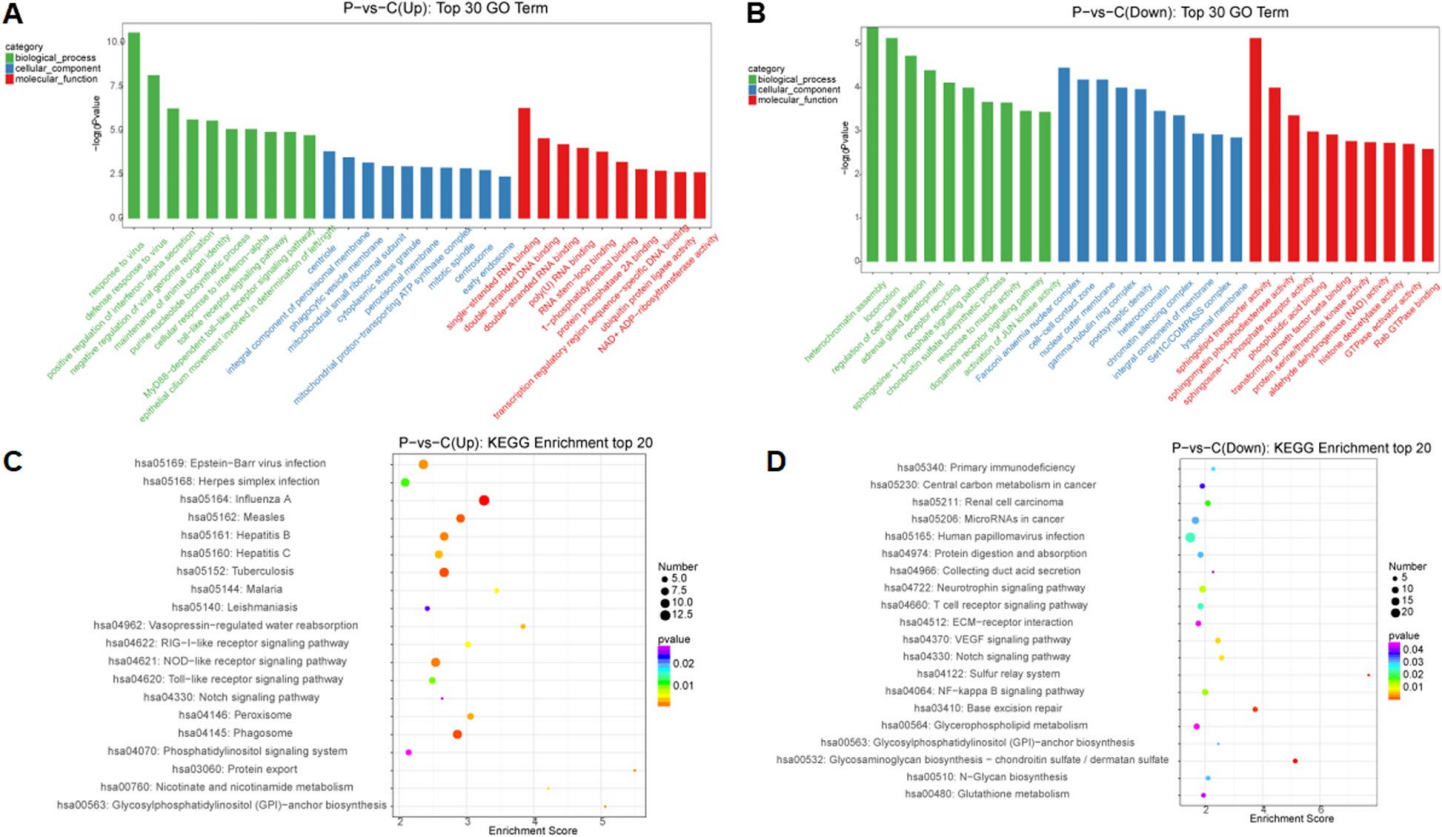
**A** GO term enrichment of the upregulated mRNAs; **B** GO term enrichment of the downregulated mRNAs; **C** KEGG pathway analysis of the upregulated mRNAs; **D** KEGG pathway analysis of the downregulated mRNAs [19, 20]

In order to further understand the function of differentially expressed genes, the GO term and KEGG pathway were analyzed. The top 30 dysregulated GO processes (biological processes, cellular components, and molecular functions) of each subgroup were analyzed based on enriched, dysregulated mRNAs derived from gene annotation. Select the prediction with a *p*-value of < 0.05 and rank them by *p*-value. Based on the GO conventional classification algorithm, the enrichment score is used to enrich the significant GO terms of differentially expressed genes. The upregulated GO enrichment of mRNA included response to virus, defense response to virus, positive regulation of interferon-alpha secretion in biological processes; centriole, integral component of peroxisomal membrane, phagocytic vesicle membrane in cellular component; and single-stranded RNA binding, double-stranded DNA binding, double-stranded RNA binding in molecular function (Fig. 3A). GO term enrichment for down-regulated mRNA included heterochromatin assembly, locomotion, and regulation of cell-cell adhesion in biological processes. Fanconi anaemia nuclear complex and cell-cell contact zone and nuclear outer membrane in cellular components; sphingolipid transporter activity, sphingomyelin phosphodiesterase activity, sphingosine-1-phosphate receptor activity in molecular function (Fig. 3B). Using KEGG pathway analysis [19, 20], upregulated mRNA is enriched in protein export, Glycosylphosphatidylinositol (GPI)−anchor biosynthesis, and Nicotinate and nicotinamide metabolism (Fig 3C), and downregulated mRNA is enriched in Sulfur relay system, Glycosaminoglycan biosynthesi−chondroitin sulfate / dermatan sulfate, and Base excision repair (Fig 3D). The results suggest that these pathways may contribute significantly to the pathogenesis of PE.

### Small RNA sequencing data analysis

**Fig. 4:**
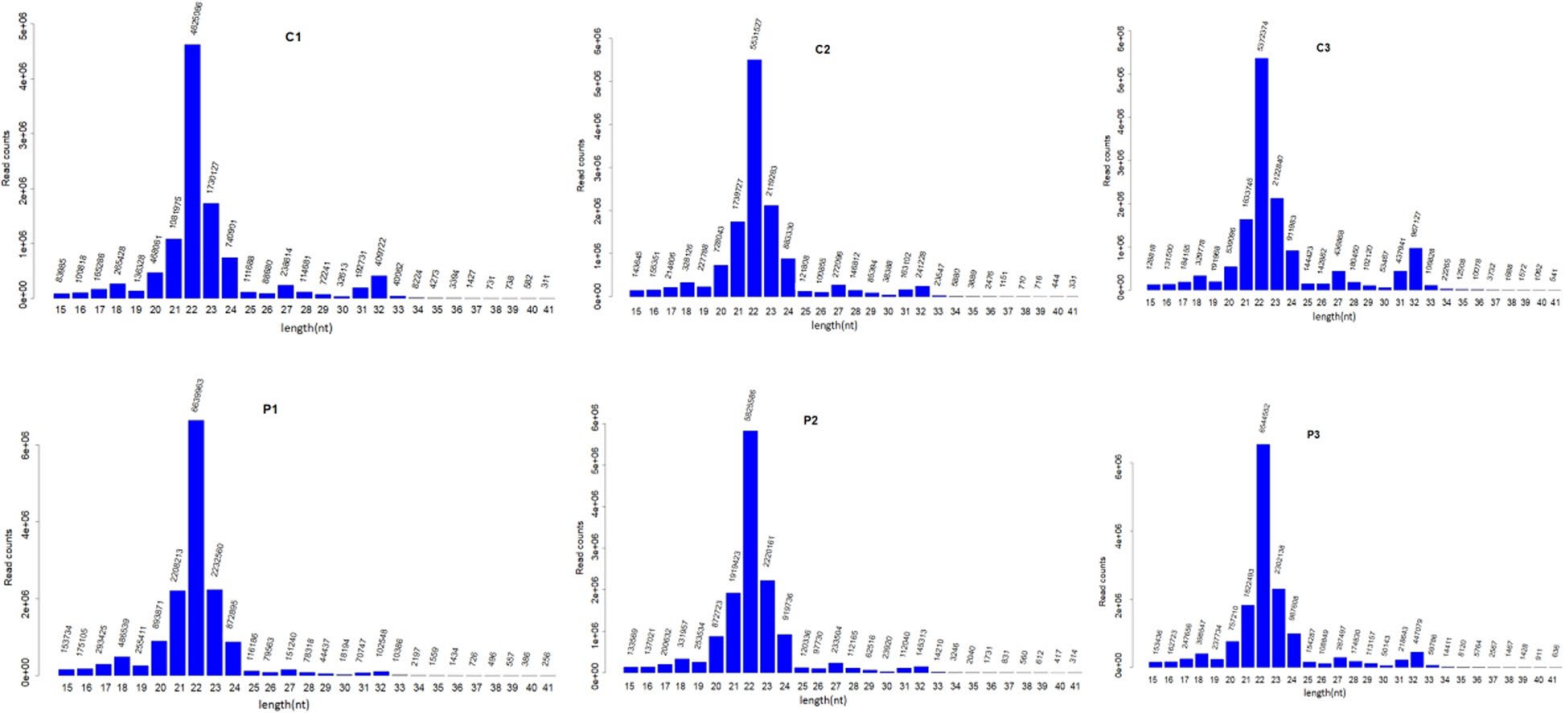
Clean reads length analysis showed that most of the clean reads were mainly distributed at 20–24 nt and 31–32 nt

To illustrate the pathogenic mechanism of preeclampsia, we first conducted a comparative transcriptomic analysis of three preeclampsia patients and three healthy individuals. Six miRNA libraries, named C1, C2 and C3 as the normal group, P1, P2 and P3 as the patient group, were constructed and sequenced. From the libraries, 11.56 M; 14.92 M; 15.22 M; 16.58 M; 15.36 M and 16.93 M clean reads were obtained (Table 3). The clean reads length analysis showed that most of the clean reads were mainly distributed at 20–24 nt and 31–32 nt (Fig. 4).

Small RNA had a great variety including microRNA, tRNA (tiRNA, tRFs), rRNA, piRNA, snRNA and so on. To classify and annotate the small RNA in the sequencing results, clean reads were compared and annotated with Rfam database, DNA sequence, species repeat sequence library, and miR base database. The results demonstrated that small RNA contained 92.42% unannotation miRNAs, 1.29% known miRNA, 5.14% repeat miRNA, 0.37% gene and 0.68% rRNA, snRNA and other Rfam RNA.

**Table 3:** the clean reads analysis of six samples(C1, C2, C3,P1, P2 and P3)

	raw_reads	reads_trimmed_length	reads_trimmed_Q20	reads_trimmed_N	clean_reads	clean_reads_uniq
C1	11.56 M	10.72 M	10.72 M	10.72 M	10.72 M	0.30 M
C2	14.92 M	13.29 M	13.28 M	13.28 M	13.28 M	0.39 M
C3	15.22 M	14.18 M	14.17 M	14.17 M	14.17 M	0.41 M
P1	16.58 M	14.90 M	14.89 M	14.89 M	14.89 M	0.38 M
P2	15.36 M	13.76 M	13.75 M	13.75 M	13.75 M	0.38 M
P3	16.93 M	15.27 M	15.26 M	15.26 M	15.26 M	0.41 M

**Fig. 5:**
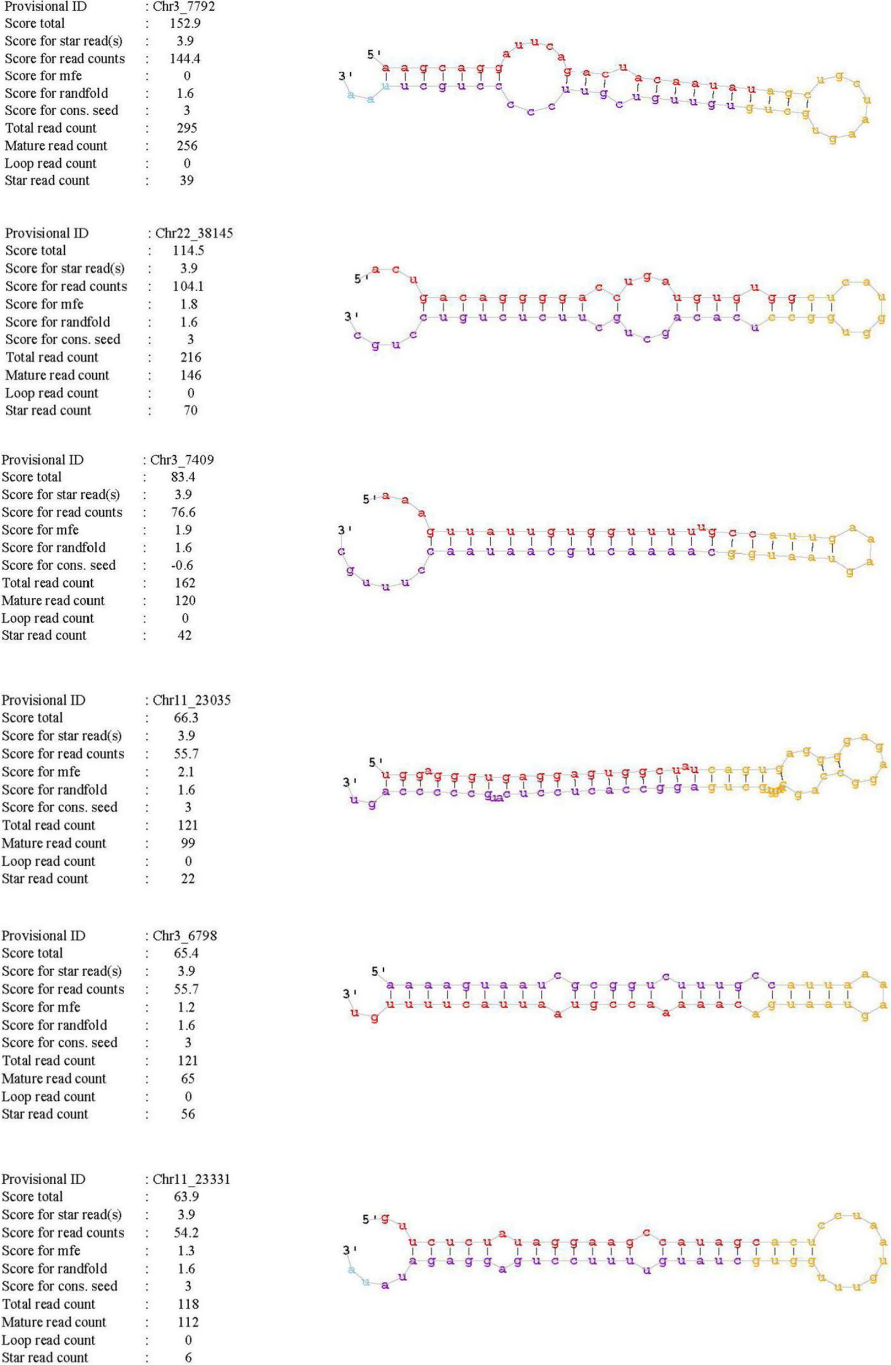
Secondary structures of six novel miRNAs were shown

Small RNA sequencing data were compared with different databases to remove small RNAs such as tRNA, snRNA and rRNA as far as possible. Then filtered sequences were compared with the Microbase database to make known microRNAs statistics. Unannotated sequences were named novel miRNAs. Next, we performed the miRDeep2 software and RNAfold software to recognize the sequence of microRNA hairpin precursors. Total 1291 and 1281 novel RNAs were obtained from the preeclampsia patients and healthy individuals. Six novel miRNAs with secondary structures were shown in the Fig. 5.

### Identification of DE miRNA

**Fig. 6:**
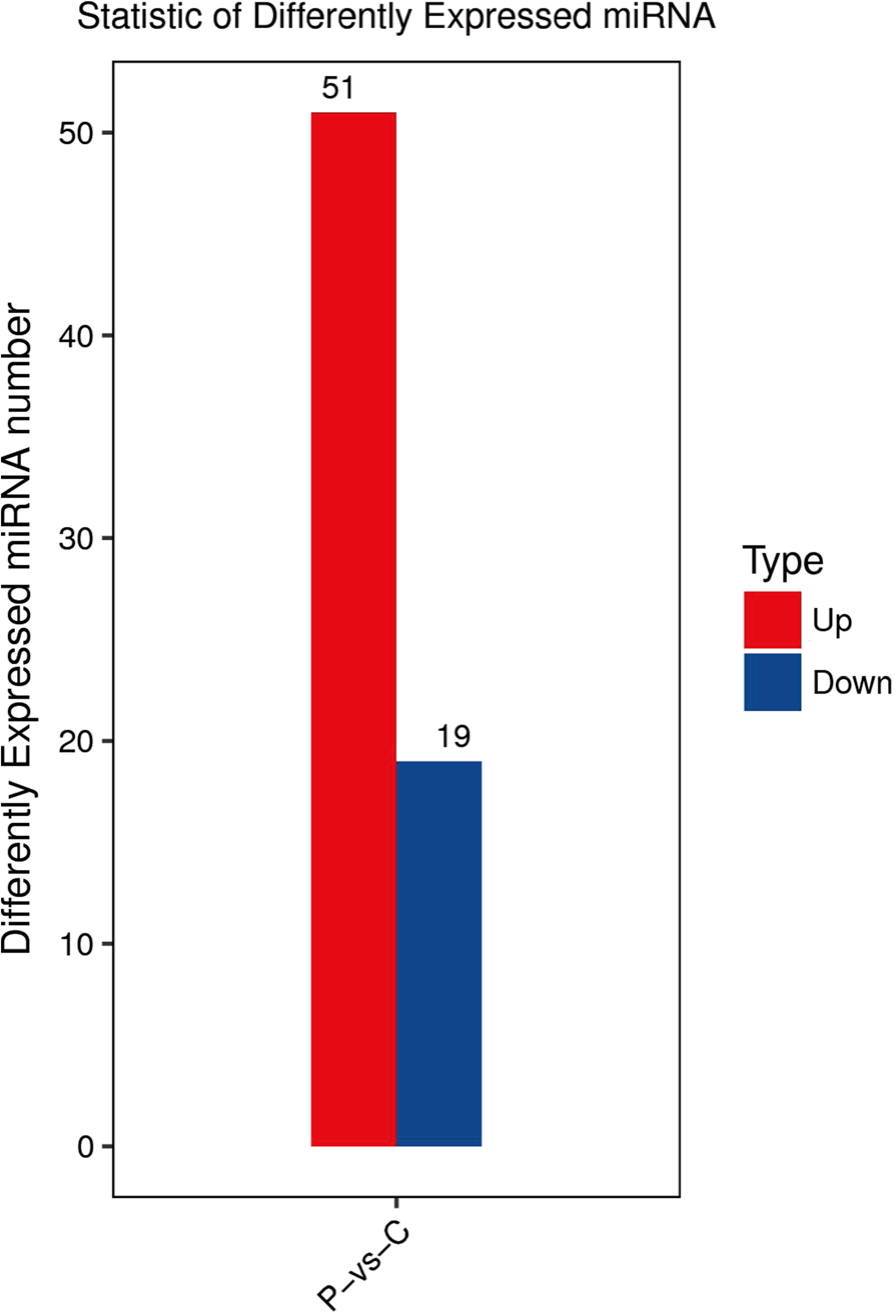
51 significantly upregulated and 19 significantly downregulated miRNAs between preeclampsia patients and healthy individuals

**Fig. 7:**
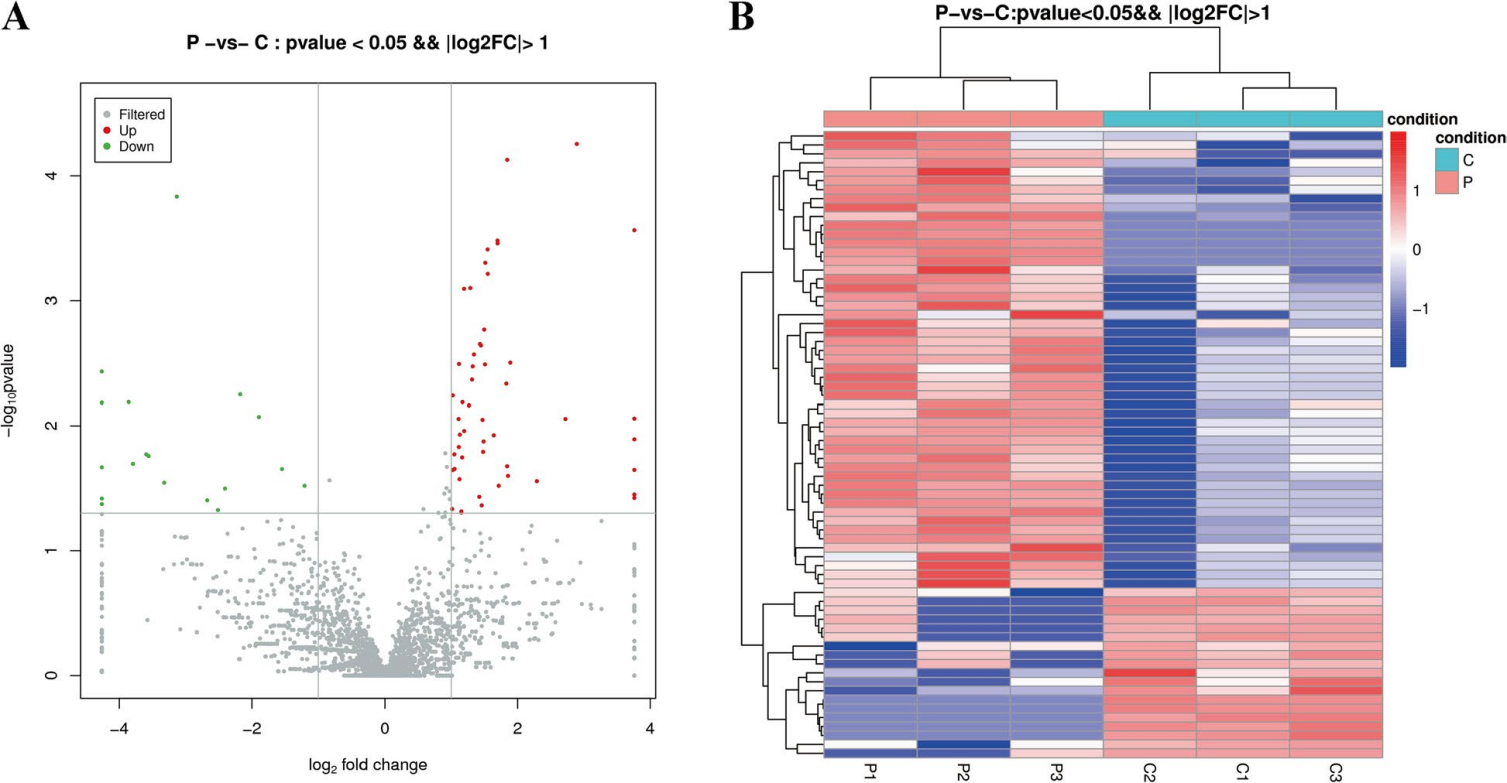
**A** volcano plots of differentially expressed microRNAs; **B** heat map of differentially expressed microRNAs

To explore the significant differences in miRNAs between preeclampsia patients and healthy individuals, the miRNAs were determined for fold change (FC > 2) and adjusted *p*-value (*p* < 0.05) by DESeq analysis. 70 miRNAs were screened out with significant levels, with 51 significantly upregulated and 19 significantly downregulated (Fig. 6). The overall distribution of DE miRNAs were mapped in volcano plots satisfying both conditions (Fig. 7A). Next a heat map was displayed by unsupervised hierarchical clustering analysis DE miRNAs (Fig. 7B), and the results confirmed that miRNA expression level could be strongly separated in individuals of preeclampsia patients from that in healthy individuals (Table 4).

**Table 4:** Screening of differentially expressed miRNAs in preeclampsia patients and healthy pregnant women

miRNA_id	baseMean	baseMean_control_C	baseMean_case_P	foldChange	log2FoldChange	pval	up_down
hsa-miR-1185-1-3p	128.8597149	69.65624963	188.0631801	2.699875188	1.432892715	0.002222962	Up
hsa-miR-1197	51.37557324	26.73629925	76.01484724	2.843132721	1.507481448	0.003239348	Up
hsa-miR-1297	7.165948891	1.886965306	12.44493248	6.595210011	2.7214186	0.008780384	Up
hsa-miR-136-3p	261.5116138	147.8966732	375.1265544	2.536409686	1.342787791	0.002698483	Up
hsa-miR-144-3p	2101.832012	1142.504687	3061.159337	2.679340726	1.421878057	0.036955607	Up
hsa-miR-154-5p	54.93581669	33.89627927	75.97535411	2.241406896	1.164404574	0.017869856	Up
hsa-miR-224-3p	5.959502679	11.31093028	0.608075078	0.053759953	-4.217324319	2.74E-05	Down
hsa-miR-299-3p	15.43374295	7.496245222	23.37124068	3.117726274	1.64049427	0.011874766	Up
hsa-miR-299-5p	15.96705364	7.01206666	24.92204063	3.554164818	1.829510585	0.004620265	Up
hsa-miR-3155a	17.77783219	11.03527558	24.5203888	2.222000587	1.151859198	0.049484458	Up
hsa-miR-3166	1.11373669	2.22747338	0	0	-Inf	0.038160536	Down
hsa-miR-3200-5p	1.215464825	2.430929651	0	0	-Inf	0.021444575	Down
hsa-miR-323a-3p	200.0306497	101.8285814	298.2327181	2.928772198	1.550295984	0.000611873	Up
hsa-miR-323b-3p	469.7541281	250.638003	688.8702532	2.748466892	1.458627101	0.043292259	Up
hsa-miR-329-3p	320.4259577	202.9039482	437.9479673	2.158400422	1.109962535	0.008784055	Up
hsa-miR-337-3p	149.9170291	94.09050125	205.7435569	2.186655977	1.128726261	0.011731975	Up
hsa-miR-337-5p	16.30358174	8.605897022	24.00126647	2.788932566	1.479713052	0.01611235	Up
hsa-miR-369-3p	1230.181372	776.6076747	1683.75507	2.168089661	1.11642442	0.003213086	Up
hsa-miR-369-5p	93.84018487	50.34162136	137.3387484	2.728135183	1.447915134	0.002288167	Up
hsa-miR-379-3p	92.15546259	43.45336913	140.857556	3.241579625	1.696697011	0.000330829	Up

### Target genes prediction of DE miRNA

To better known the potential roles of 70 DE miRNAs, miRanda algorithms was used to predict and analyze the target genes. The results suggested that 44,306 genes were predicted as the targets of these miRNAs.

### GO term & KEGG pathway enrichment analysis of DE miRNA

**Fig. 8:**
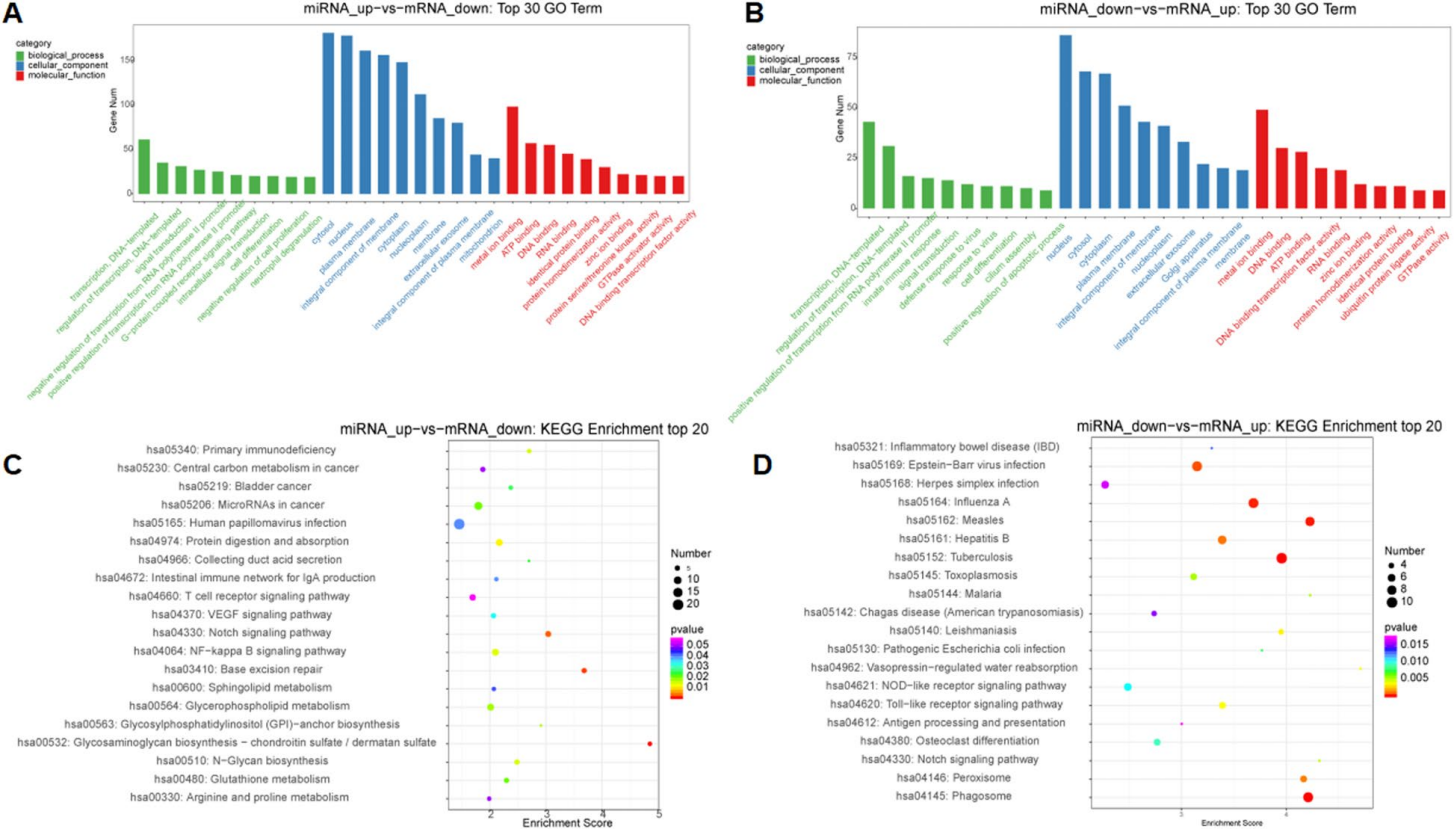
**A** GO term enrichment of the upregulated miRNAs; **B** GO term enrichment of the downregulated miRNAs; **C** KEGG pathway analysis of the upregulated miRNAs; **D** KEGG pathway analysis of the downregulated miRNAs [19, 20]

To further elucidate the function of DE genes, GO term and KEGG pathway analyses were applied respectively. According to GO classification system, miRNAs were classified into biological process, cellular component, and molecular function. Top 30 GO processes for each subgroup were analyzed according to enriched. GO term enrichment of the upregulated miRNAs included transcription, DNA-templated; regulation of transcription, DNA-templated; signal transduction; cytosol; nucleus; plasma membrane; metal ion binding; ATP binding; ATP binding (Fig. 8A). GO term enrichment of the downregulated miRNAs included transcription, DNA-templated, regulation of transcription, DNA-templated, positive regulation of transcription from RNA polymerase II promoter, nucleus, cytosol, cytoplasm, metal ion binding, DNA binding, ATP binding (Fig. 8B). In the results of the KEGG pathway analysis [19, 20], the upregulated miRNAs were enriched in Glycosaminoglycan biosynthesis-chondroitin sulfate / dermatan sulfate, Base excision repair (Fig. 8C), and the downregulated miRNAs were enriched in Tuberculosis, Phagosome (Fig. 8D). The results suggest that these pathways may have a significant contribution to the pathogenesis of preeclampsia.

### Integrated analysis of miRNA-mRNA interaction network

**Fig. 9:**
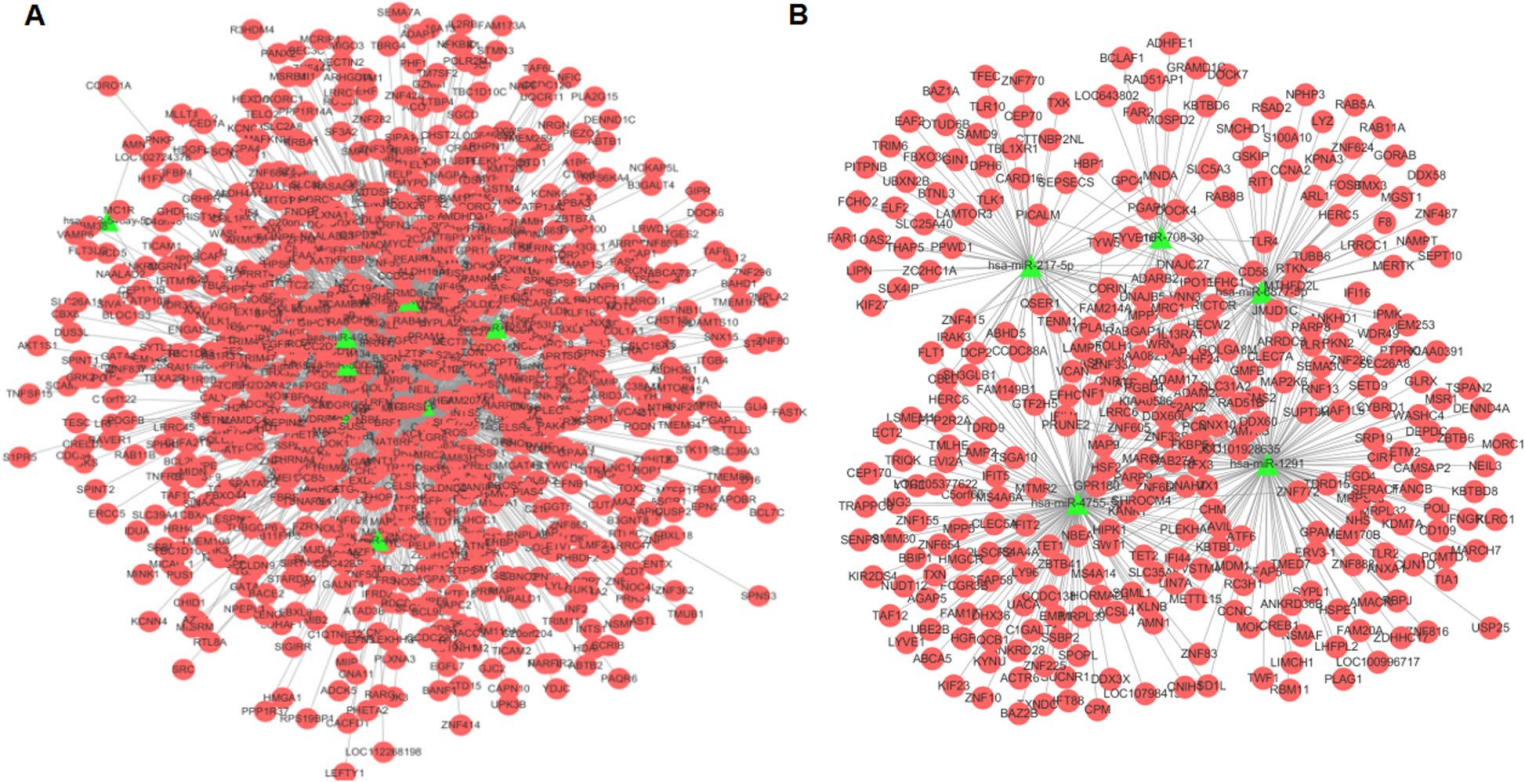
**A** a network of negatively correlated upregulated miRNAs and downregulated mRNAs; **B** a network of downregulated miRNAs and upregulated mRNAs

To understand the mechanisms of miRNA regulation in the pathogenesis of preeclampsia, we investigated the interactions between upregulated or downregulated miRNAs and their target downregulated or upregulated mRNAs, respectively. Based on negatively correlation, the regulatory network was constructed by upregulated miRNAs and downregulated mRNAs (Fig. 9A) and another regulatory network was constructed by downregulated miRNAs and upregulated mRNAs (Fig. 9B). There were 2208 negative miRNA-mRNA interactions in total. The regulatory networks of differentially expressed miRNAs(hsa-miR-1255a, hsa-miR-3155a, hsa-miR-3161,hsa-miR-4489, hsa-miR-494-3p, hsa-miR-5000-3p, hsa-miR-1291, hsa-miR-217-5p, hsa-miR-4755-5p, hsa-miR-6877-5p, hsa-miR-708-3p) and the mRNAs they regulate are shown in the figure.

## Discussion

Preeclampsia plays a key role in maternal and perinatal morbidity in all world [8]. Respectively, before and after 34 weeks of gestation are called early and late onset preeclampsia [34]. The molecular mechanisms of PE pathogenesis are complex and multifactorial processes influenced by many factors [2, 30]. Accumulating evidence shown significant connection between PE and the later risk for hypertension, atherosclerosis, ischemic heart disease, congestive heart failure, stroke, deep venous thrombosis and metabolic syndrome [23]. Therefore, it is critical to study the mechanism to find out novel reliable and effective molecular targets for curing PE.

MiRNAs, as novel regulators, have observed that play critical roles in various biological processes including inflammation cancer and infectious diseases [27]. They regulate the expression of level of protein-coding genes in a post-transcriptionally manner [17]. Accumulating evidence has indicated that miRNAs are dysregulated in placentas of patients with PE [36]. Thus, to comprehend the combined analysis of the miRNAs-mRNA network may help improve a new insight to therapy against PE patients.

Although amount of circulating microRNAs in maternal blood leukocytes of the patients with preeclampsia have reported [15], the mechanisms of miRNA-mRNA interactional regulatory in the process of PE are less identified. In our present study, the miRNA expression pattern in PE patients and healthy individuals were identified by small RNA sequencing. Total 1291 and 1281 novel RNAs were obtained from the preeclampsia patients and healthy individuals. 2565 miRNAs with significant levels of differential expression, with 51 significantly upregulated and 19 significantly downregulated. It is maybe an important reason to alter the number of miRNAs disparity that the time-space specific expression of miRNAs [6].

Then we researched DE miRNAs from PE, compared with those from healthy women. Earlier study has showed miRNA over-expression (miR-215, miR-155, miR-650, miR-210, miR-21) and under-expression (miR-18a, miR-19b1) were closely related to PE [9, 17, 22, 24, 27]. Other miRNAs such as miR-193b-3p/5p, and miR-365a/b-3p have also been previously confirmed in studies measuring miRNA expression in PE pregnancies [17, 35, 38]. These results demonstrated the possibility that miRNAs are likely to participate in PE pathogenesis.Collectively, through the miRNA-mRNA interaction network, we indicated that these differentially expressed miRNA (hsa-miR-1255a, hsa-miR-3155a, hsa-miR-3161,hsa-miR-4489, hsa-miR-494-3p, hsa-miR-5000-3p, hsa-miR-1291, hsa-miR-217-5p, hsa-miR-4755-5p, hsa-miR-6877-5p, hsa-miR-708-3p) may play crucial roles in the progress of PE pathogenesis by regulating the expression level of target genes.

Increasing evidence has indicated the key role of miRNAs in the modulation of gene expression [4]. Our predictions of 70 differentially expressed miRNA-expressing target genes revealed 44,306 putative target genes. Hsa-miR-494-3p/Notch-1, hsa-miR-494-3p/HIF1AN, hsa-miR-217-5p/EGR1, hsa-miR-217-5p/ATXN7L1, hsa-miR-217-5p/TXNRD2 regulatory axes deserve our attention. Ou’s research confirm circ_0111277 sponged hsa-miR-494-3p in trophoblast cells to regulate HTRA1/Notch-1 expression in PE [25]. This is consistent with our results of hsa-miR-494-3p’s differential expression in the blood. Hsa-miR-494-3p may influence the development of preeclampsia disease by affecting the expression of Notch-1. Yang’s study proved that hsa-miR-217-5p ameliorates inflammatory damage of endothelial cells induced by oxidized LDL by targeting EGR1. Tian’s work demonstrates a non-monotonic association between selenium concentration and expression of miR-216a-5p/miR-217-5p clusters [31]. Previous studies have confirmed that selenium has a strong relationship with the onset of preeclampsia. The miRNA-mRNA pairs identified in this study provide important clues to the pathogenesis of preeclampsia.

To comprehensively researched the functions of these DE miRNAs in the process of PE. GO enrichment and KEGG pathways analysis for differentially expressed mRNA and targets of differentially expressed miRNA were performed. Some important signaling pathways were included, such as Notch signal pathway and VEGF signaling pathway. Fragkiadaki’s study found that mRNA expression of receptors NOTCH2 and NOTCH3 and their ligands DLL3, DLL4, JAG1 and JAG2 in PE placenta is reduced. NOTCH1, NOTCH2, JAG1, and DLL4 defects result in failure to integrate the placental arterial vasculature into the maternal circulation. They suggest that Notch pathway downregulation is associated with PE [11, 32]. Previous studies have used VEGF inhibitors to treat hypertension, proteinuria, glomerular endothelial injury, elevated circulating liver enzymes, cerebral oedema, and other related conditions, similar to those of PE and eclampsia in humans. These studies point to the central role of sFLT1 and impaired VEGF signaling in PE development [28]. In mammalian melanocytes, the interaction network between miRNAs, target genes, and transcription factors plays a role in the balance of gene expression [1].

## Conclusions

Taken together, our findings reveal new insights into the pathogenesis of PE. Network and pathway information illustrates the potential function of mRNAs and miRNAs in the pathogenesis of PE. However, the specific molecular mechanisms of these imbalanced miRNAs and mRNAs in PE are not elaborated here. Future studies are needed to illustrate the underlying mechanisms of these dysregulated mRNAs and miRNAs in PE pathogenesis.

## Data Availability

The GEO dataset data used in this study are available in the GEO database. Raw data of whole transcriptome sequencing has been uploaded to Sequence Read Archieve: PRJNA665923. Small RNA sequencings data has been uploaded to Sequence Read Archieve: PRJNA836097.
